# Scalable Air-Tolerant μL-Volume Synthesis of Thick Poly(SPMA) Brushes Using SI-ARGET-ATRP

**DOI:** 10.1021/acsapm.3c01628

**Published:** 2023-08-24

**Authors:** Lars B. Veldscholte, Sissi de Beer

**Affiliations:** † Functional Polymer Surfaces Department of Molecules & Materials MESA+ Institute for Nanotechnology, University of Twente, P.O. Box 217, 7500 AE Enschede, The Netherlands

**Keywords:** polymer brushes, ATRP, RDRP, SI-ATRP, ARGET

## Abstract

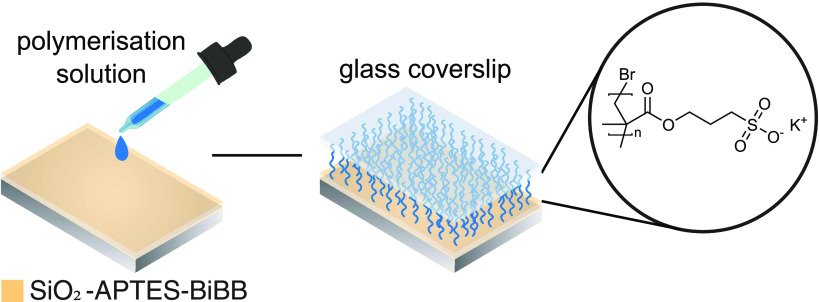

We present a facile procedure for preparing thick (up to 300 nm) poly(3-sulfopropyl methacrylate) brushes using SI-ARGET-ATRP by conducting the reaction in a fluid film between the substrate and a coverslip. This method is advantageous in a number of ways: it does not require deoxygenation of the reaction solution, and the monomer conversion is much higher than usual since only a minimal amount of solution (microliters) is used, resulting in a tremendous reduction (∼50×) of wasted reagents. Moreover, this method is particularly suitable for grafting brushes to large substrates.

## 1 Introduction

Polymer brushes are coatings consisting of macromolecules end-grafted to a surface at densities sufficiently high that the chains are forced to stretch away.^1−3^ They can be used in a broad range of applications, from sensors^4,5^ to lubricants^6,7^ and antifouling surfaces,^8,9^ in liquid and in air.^10^ There are two general ways of creating polymer brushes: *grafting-from*,^3,11^ in which polymers are grown from an initiator-functionalized surface, and *grafting-to*,^12−14^ in which presynthesized polymers are immobilized on a surface. Grafting-to is generally easier to perform and allows better control over the chain length (distribution) but suffers from a drawback: attainable grafting densities are typically low as steric hindrance of already grafted chains precludes the grafting of additional chains.

Polymer brushes can be prepared in a grafting-from manner using surface-initiated reversible-deactivation radical polymerizations (RDRPs, also called controlled radical polymerizations),^15,16^ such as atom-transfer radical polymerization (ATRP),^17,18^ reversible addition–fragmentation chain-transfer polymerization (RAFT),^19,20^ and nitroxide mediated polymerization (NMP)^21^. Of these, ATRP is the most widely utilized method, owing to its compatibility with a wide range of monomers, good control, and experimental accessibility.^11,16^ However, conventional ATRP has a couple of drawbacks, most notably the fact that it is oxygen-sensitive and thus has to be carried out under anaerobic conditions. This complicates the synthesis of polymer brushes using ATRP significantly in a few ways. Most importantly, it requires the use of airtight reaction vessels and rigorous deoxygenation of reaction solutions (e.g., by purging with an inert gas like nitrogen). Even small amounts of oxygen inadvertently introduced into the system (e.g., by inadequate deoxygenation of a reactant or an air leak) will result in termination of the polymerization reaction. Moreover, it means scaling up to larger surfaces is difficult, since equally large airtight glassware (to contain them) is required, and conversion is extremely low; only a small fraction of monomer in the solution is polymerized on the surface.^22,23^

Air-tolerant polymerization methods greatly simplify polymer brush synthesis^9,24,25^ and make it accessible to more people, as well as much more convenient for experienced chemists. Over the past few years, many developments in air-tolerant SI-ATRP (most of which are based on activator regeneration)^22,23,26^ as well as in air-tolerant SI-RAFT^27−29^ have taken place.

In ATRP, the equilibrium between propagating and dormant chain ends is mediated by transfer of a halide to a transition metal catalyst (usually copper).^17,30^ The mechanism by which oxygen interferes with ATRP is by oxidizing the Cu(I)-ligand complex (the activator species) to Cu(II), as well as by quenching the propagating radicals, although the former mechanism dominates over the latter at ATRP equilibrium because of the low concentration of propagating chain ends. Activator regeneration methods are based on continuously *re*generating the activator species by steadily reducing Cu(II) back to Cu(I). In activators regenerated by electron transfer (ARGET), this is achieved using an excess of a slowly reacting nonradical forming reducing agent. In this way, the system formed by the Cu catalyst and reducing agent acts as a kind of oxygen scavenger. The same mechanism also allows for a tremendous reduction of catalyst concentrations, to (sub-)100 ppm levels (relative to monomer): although in principle only the *ratio* of Cu(I) to Cu(II) affects the polymerization kinetics, in reality with normal ATRP a rather large absolute quantity of catalyst is required as a buffer, since inevitable chain termination reactions irreversibly convert Cu(I) to Cu(II). With ARGET, accumulated Cu(II) is continuously reduced back to Cu(I). (22,30,31) Note that activator regeneration methods only render ATRP oxygen tolerant to a limited extent since the rate of oxygen diffusion into the system should not exceed the activator regeneration rate. This unfortunately means conducting ATRP wholly open to air is still not feasible, since in that case, the rate of oxygen diffusion typically exceeds the activator regeneration rate. However, these methods do enable conducting ATRP without the need to deoxygenate the solution, as long as measures are taken to limit fresh air exchange during the polymerization, for example, by using sealed vessels like capped vials or jars.^22^

Instead of using milliliters of solution in sealed vessels, the reaction volume can be reduced by conducting the reaction in a fluid film between the substrate and a coverslip employed as an oxygen barrier.^32^ This significantly reduces the amount of wasted monomer and other reagents and facilitates scalability. Recently, Flejszar et al. reported a procedure for polymerizing 2-(dimethylamino)ethyl methacrylate (DMAEMA) using SI-ARGET-ATRP under a coverslip to limit oxygen exposure.^33^ DMAEMA is special as a monomer for ARGET-ATRP because it itself acts as a reducing agent,^34^ eliminating the need for a dedicated one.

In this work, we generalize Flejszar et al.’s method of SI-ARGET-ATRP under a coverslip by adapting it to 3-sulfopropyl methacrylate (SPMA), an anionic monomer that does not have intrinsic reducing properties. As such, we needed to use a dedicated reducing agent. Like poly(DMAEMA), poly(SPMA) is a strongly hydrophilic polyelectrolyte that has applications in antifouling and antibacterial surfaces,^35−39^ reverse osmosis membranes,^40^ lubricious surfaces,^41−44^ catalysts,^45^ and reversible protein adsorption.^46^ However, poly(DMAEMA) is a weak polybase (p*K*_a_ = 7.5^47,48^ that is only charged under acidic conditions, which limits the applicable pH range when high charge density is desired. In contrast, poly(SPMA) is a strong polyacid (p*K*_a_ < 3^49,50^) that is almost fully charged under moderate conditions.

**Figure 1:**
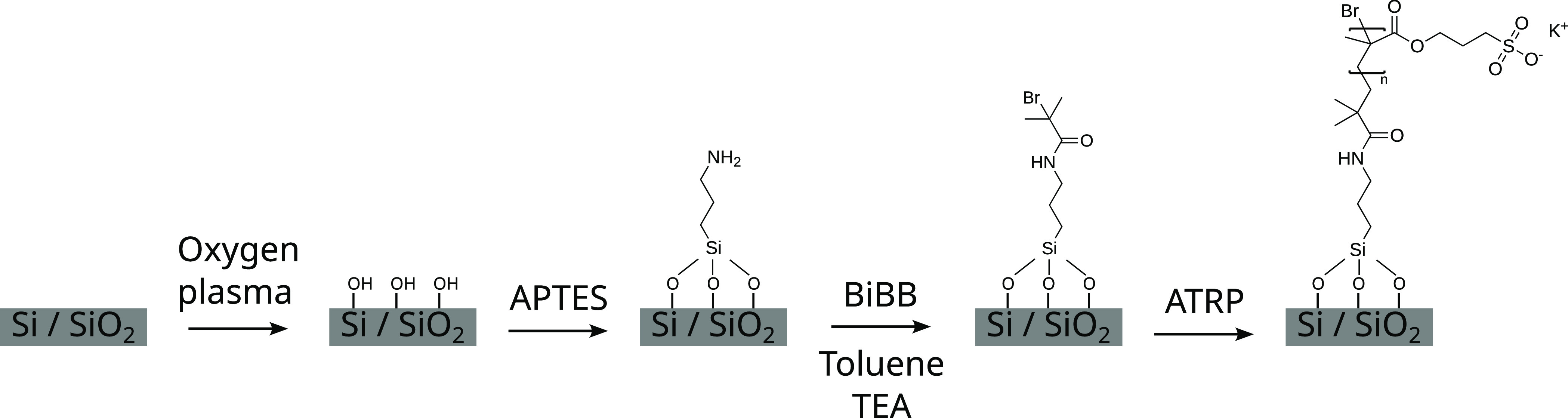
Schematic overview of the surface modification steps leading up to the polymer brush: plasma cleaning, anchor deposition, initiator coupling, and finally polymerization.

We present the process and results of adapting and optimizing this method for poly(SPMA), and we discuss the nontrivial influence of the reducing agent concentration on polymerization kinetics in this configuration. The Design of Experiments (DoE) principle is employed to systematically vary various parameters, with the goal of determining their effects and, ultimately, finding the optimal conditions. The brushes are grafted on silicon wafers as a model substrate. To this end, the wafers are first decorated with a silane anchor (APTES) and an ATRP initiator (BiBB), providing surface-bound initiation sites (Figure 1). This is an established procedure described previously.^51^

## 2 Materials and Methods

### Materials

Potassium 3-sulfopropyl methacrylate (SPMAK, 98%), (3-aminopropyl)triethoxysilane (APTES, 98%), αbromoisobutyryl bromide (BiBB, 98%), 2,2′-bipyridine (BiPy, 98%), triethylamine (TEA, 98%), ascorbic acid (AA), copper(II) chloride (CuCl_2_), toluene (99.5%), and methanol (99.8%) are purchased from Merck and used as received.

Silicon wafers (o.d. 10 cm, 525 μm thick, boron-doped with a (100) crystal lattice orientation) are purchased from Okmetic.

### Preparation of Initiator-Functionalized Silicon Substrates

Silicon wafers are cut into pieces, rinsed with water and ethanol, and dried under a nitrogen stream. The substrates are cleaned and activated by plasma cleaning with oxygen plasma for 20 min and subsequently functionalized by vapor deposition of APTES (0.1 mL) in a desiccator under a vacuum overnight. Next, they are rinsed with water and ethanol and dried again, and the initiator (BiBB) is coupled by reaction in a solution consisting of 100 mL of toluene, 1 mL of TEA, and 1 mL of BiBB for 3 h. The reaction mixture is vigorously stirred to prevent settling of aggregates on the substrates. After the reaction, the samples are thoroughly rinsed with water and ethanol and dried once again.

### Surface-Initiated Polymerization of SPMA by ARGET-ATRP

A stock solution (“ATRP cocktail”) containing the monomer (SPMAK), copper halide salt (CuCl_2_), and ligand (BiPy) in 4:1 water to methanol is prepared. The concentrations are systematically varied to determine optimal conditions, as described in the results section. Another stock solution of 50 mM ascorbic acid in water is made. The latter solution has to be prepared fresh daily unless stored under anaerobic conditions since aqueous solutions of ascorbic acid are not stable in aerobic conditions.

The initiator-functionalized substrates are placed on a flat surface and a desired volume (typically 100 μL per substrate of 1 cm^2^) of “ATRP cocktail” is mixed with an amount of ascorbic acid stock solution in an Eppendorf tube. An immediate color change from very light blue to light orange confirmed the reduction of Cu(II) to Cu(I). After pipet mixing, the drops of the solution are deposited on the substrates and covered with a glass coverslip, taking care not to trap any air bubbles, as these will create local defects in the resulting brush. As the solution becomes oxygen sensitive after mixing in ascorbic acid, the solution has to be deposited and covered rapidly, as to avoid oxidation by environmental oxygen. The substrates are covered with a Petri dish to minimize air currents and left to polymerize for the desired amount of time. To terminate the polymerization, the coverslips are removed, and the samples are rinsed with water and ethanol and dried.

### Determination of Brush Thickness Using Ellipsometry

The (dry) thickness of brushes is determined using a Woollam M-2000X variable angle spectroscopic ellipsometer (VASE). Measurements are performed at angles of 65°, 70°, and 75° and at wavelengths between 300 and 1000 nm.

The ellipsometric data are fitted using the CompleteEASE software to a model composed of a Si substrate, a 1 nm native oxide layer, and a Cauchy layer for the polymer brush. This topmost layer’s thickness and Cauchy A and B parameters are fitted. We do not use higher-order Cauchy coefficients, and we assume that the film is transparent over the measured wavelength range.

### Determination of Brush Thickness and Surface Morphology Using Atomic Force Microscopy

Samples were measured by using a Bruker MultiMode 8 atomic force microscope in PeakForce QNM mode with an Olympus OMCL-AC240TS cantilever. To determine the film thickness, a scratch in the brush was made using a steel needle.

## 3 Results and Discussion

Kim et al. presented a recipe for the surface-initiated polymerization of various monomers including SPMA using ARGET-ATRP (in a closed vial, without deoxygenation),^52^ which is used as a starting point (Table 1). Here, a catalyst concentration of 100 ppm is used, and the ligand is present in 6× excess to the copper. The solvent is a 4:1 mixture of water and methanol. Kim et al. report around 90 nm thick brushes after 4 h using this recipe.

**Table 1:** Recipe by Kim et al.^52^

SPMA (mM)	CuBr_2_ (mM)	BiPy (mM)	Ascorbic acid (mM)
620	0.063	0.38	3.1

We reproduced this recipe (in a closed vial with 3.3 mL solution) with some small changes: CuCl_2_ instead of CuBr_2_, and a 10× instead of a 6× excess of ligand. The former is expected to slightly improve control,^53^ and a substantial excess of ligand to copper is known to be beneficial in aqueous ARGET-ATRP, because in water the Cu(I)/ligand complex is liable to dissociate. An excess of ligand shifts the equilibrium toward the Cu(I)/ligand species.^54^ At 4 h polymerization, we obtain 140 nm thick brushes, and a fairly linear thickness-overtime relationship between 2 and 6 h (see Supporting InformationTable S2).

PMDETA instead of BiPy as a ligand was also tried, but this only yielded thin brushes (no thicker than 41 nm after 4 h, see Supporting InformationTable S3) and uncontrolled polymerization (nonlinear thickness over time). It is not completely clear why the polymerization is so poorly controlled with PMDETA, but it is mentioned in literature that the Cu(II)/PMDETA complex is unstable toward protonation, which could be problematic in ARGET as protons are released as a side product of the oxidation of the reducing agent.^30^

**Figure 2:**
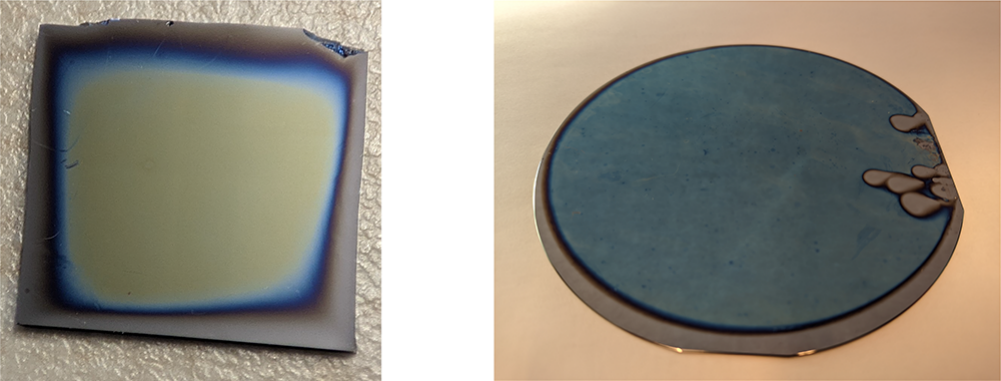
Left: poly(SPMA) brush on a 1 cm^2^ piece of silicon. Note the thickness gradient at the edges, which occurs due to oxygen diffusion. Right: an entire o.d. 10 cm silicon wafer grafted with poly(SPMA). The “holes” are due to entrapment of air bubbles at those positions. This wafer also spots the same thickness gradient at the edge, although to a relatively smaller extent due to the increased size of the wafer.

Entries 1–3 in Table 2 show the results of a first experiment using this modified recipe (and a higher and lower reducing agent concentration) performed under a coverslip. This successfully resulted in homogeneous brushes with the exception of a notable thickness gradient of a few millimeters wide around the edges, caused by oxygen diffusion from the surrounding air (see Figure 2). This “edge effect” has been noted by others performing air-tolerant SI-ATRP in a liquid film sandwiched between the substrate and a cover.^23,32^ The reducing agent concentration was varied first, as that parameter is expected to be most critical when changing the setup (and thus the amount of oxygen diffusion). Predicting the optimal amount of reducing agent is not trivial: too much will produce too much activator (Cu(I)) in the beginning of the polymerization reaction, leading to overly fast and poorly controlled ATRP. On the other hand, an insufficient amount of reducing agent results in poor oxygen tolerance, and thereby too slow polymerization (or none at all).^26^ Both cases result in thin brushes. This experiment clearly shows that 10 mM ascorbic acid is excessive since it results in thinner brushes than are obtained with lower concentrations.

**Table 2:** Initially Tried Recipes for Polymerization Were Tried under a Coverslip

				Thickness (nm)	
#	[M]^b^ (mM)	[Cu]^c^ (mM)	[RA]^d^ (mM)	2 h	4 h
1	620	0.063	3.1	141	98^a^
2	620	0.063	1.5	115	123
3	620	0.063	10.0	62	76
4	1000	0.200	2.0	220	37^a^

a These samples were inhomogeneous and therefore measured thinner by ellipsometry.

b Monomer (SPMA) concentration.

c Copper catalyst concentration.

d Reducing agent (ascorbic acid) concentration.

Next, a full factorial design was performed with three factors (monomer concentration, catalyst concentration, and reducing agent concentration) and two levels, i.e., a 2^3^ design. From this, it was identified that the higher monomer concentration (1 M), higher catalyst concentration (0.2 mM), and lower reducing agent concentration (2 mM) produced the thickest brushes (no. 4 in Table 2). The full results are available in Supporting Information (Table S1).

**Figure 3:**
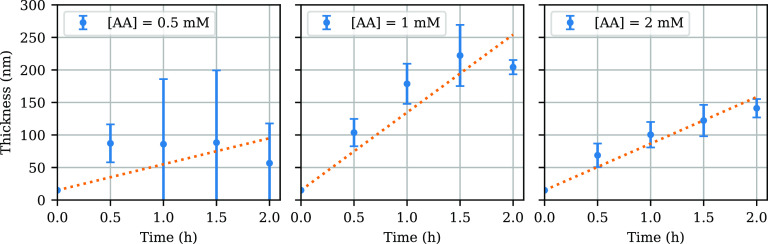
Kinetics for several values of the reducing agent concentration with 90% confidence intervals and linear fits. A linear increase in thickness over time is expected in the case of good polymerization control. For the lowest reducing agent concentration of 0.5 mM, brushes with wildly fluctuating thicknesses are obtained because of the insufficient oxygen tolerance. The sample size per point is 4, 7, and 5, respectively, for the brushes with 0.5, 1, and 2 mM of reducing agent.

Lowering the reducing agent concentration further to 1 mM yielded even thicker brushes (∼180 nm after 1 h), indicating that we are still in the regime of “overly fast ATRP”. However, when the reducing agent concentration was again cut in half to 0.5 mM, brushes with inconsistent thicknesses (∼86 nm after 1 h) and large edge gradients were obtained (Supporting Information, Section S6). Likely, 0.5 mM of ascorbic acid does not provide sufficient oxygen tolerance, making the process too susceptible to small variations in oxygen ingress and thereby yielding inconsistent results. Therefore, ARGET-ATRP in this configuration presents a trade-off between oxygen tolerance and polymerization control; in contrast to ATRP performed under anaerobic conditions, lowering the reducing agent concentration does not per se lead to better control over thickness. The results of the kinetic study for several reducing agent concentrations are listed in Figure 3.

This method was successfully applied to larger substrates, such as whole 10 cm Si wafers (Figure 2, right). Instead of covering them with a coverslip, two equally sized substrates can simply be sandwiched together, sharing the liquid film of polymerization solution between them. This further increases the efficiency of the process, as only half the amount of reagent per area polymer brush is required. Two identical brushes are obtained, with no differences between the bottom and top wafers (beyond differences in initiator density; see Supporting Information, Section S7).

Thick (>200 nm) brushes sometimes developed a hazy, rough finish (see Supporting Information, Section S5). It was not possible to remove this haze by rinsing or ultrasonic cleaning without also completely degrafting the brush. However, using a more resilient APTES-PGMA-TRIS grafting layer^55,58^ instead of APTES permitted the removal of the haze without degrafting the brush. Although we were not able to ascertain the exact mechanism behind the emergence of the surface roughness, we postulate that it is caused by nongrafted polymer that entangles with the brush at sufficiently high chain lengths. Moreover, we found that the issue could be prevented by more thoroughly cleaning the wafers after initiator coupling with ultrasonic cleaning for 5 min in an ethanol/water mixture. This suggests that surplus physisorbed BiBB that is present on the wafers when they are not adequately cleaned is the source of nongrafted polymer. Note that even in the case this nongrafted polymer is able to be removed, it will reduce the thickness of the resulting brush since these improperly grafted chains will sterically hinder growth of neighboring chains without contributing to the final grafting density.

**Figure 4:**
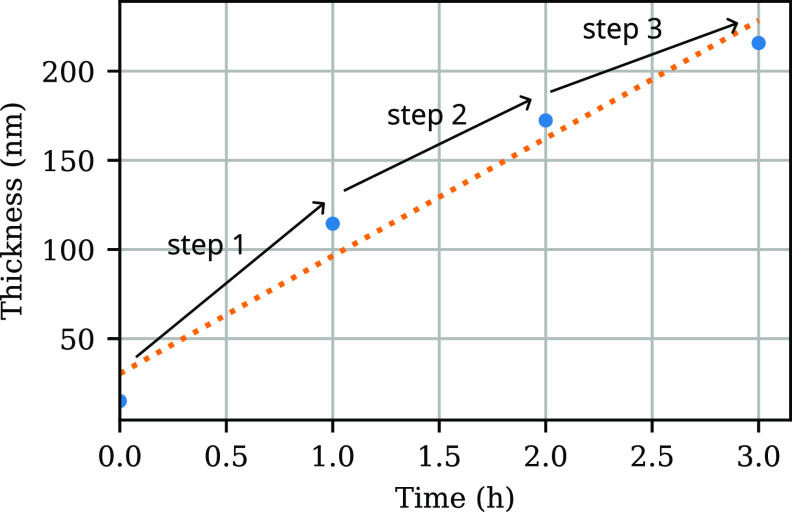
Thickness of a polymer brush grown in multiple steps (chain extension).

Finally, retention of chain-end functionality was confirmed by a chain extension experiment in which the same wafer is polymerized in multiple steps, and its thickness is measured in between (Figure 4). The procedure is the same as outlined before, except a sample already containing a brush is used instead for the subsequent steps. This technique can also be used to produce block copolymer brushes by using different monomers for each step.

The successful, homogeneous formation of well-defined poly(SPMA) brushes was confirmed by FTIR spectroscopy, AFM, and optical microscopy (Supporting Information, Sections S2–S4).

## 4 Conclusion and Outlook

In summary, we presented an air-tolerant SI-ARGET-ATRP synthesis of poly(SPMA) brushes on silicon wafers that does not require any deoxygenation or an inert atmosphere by performing the polymerization under a coverslip as an oxygen barrier. This results in a tremendous reduction in (wasted) reagents and required glassware and facilitates scaling up to large surfaces.

In particular, we investigated the brush growth kinetics as a function of the reducing agent concentration, which showed a nontrivial influence: while the reducing agent concentration controls the polymerization speed in ARGET-ATRP, in this configuration it also counteracts the effects of oxygen. The fastest brush growth was observed with 1 mM of ascorbic acid, which yielded a growth rate of 120 nm h^–1^ on average. This method was successfully applied to an entire 10 cm silicon wafer.

Although the presence of oxygen in the system complicates ARGET-ATRP, we believe the advantages of this method outweigh the drawbacks considerably.

One of the drawbacks of this method, the formation of a thickness gradient along the edge, can potentially be prevented by sealing off the edges. For example, one can envision conducting the polymerization in a cell in which the coverslip (or another lid) fits perfectly in such a way that it is enclosed from all directions instead of just from the top.

## Supporting Information Available

Full results of DoE experiments, IR spectra, AFM images, optical microscopy images, and photographs (PDF)
