# Supplementary material for: Scalable Air-Tolerant μL-Volume Synthesis of Thick Poly(SPMA) Brushes Using SI-ARGET-ATRP
Source: ACS Appl Polym Mater. 2023 Aug 24;5(9):7652–7. doi: 10.1021/acsapm.3c01628 (PMC10496111; doi:10.1021/acsapm.3c01628)
Supplement: Supplementary file 1 — ap3c01628_si_001.pdf [file ap3c01628_si_001.pdf]

Scalable air-tolerant µL-volume synthesis of thick poly(SPMA) brushes using SI-ARGET-ATRP: Supporting information

Lars B. Veldscholte[1,*] and Sissi de Beer[1]

[1]Functional Polymer Surfaces, Department of Molecules & Materials, MESA+ Institute for Nanotechnology, University of Twente, P.O. Box 217, 7500 AE Enschede, the Netherlands
[*]Corresponding author: l.b.veldscholte@utwente.nl

2023-08-17

S1 Initial results

Table S1: First 2^3 DoE experiment

#	[M] (mM)	[Cu] (mM)	[RA] (mM)	Thickness (nm)	
				2 h	4 h
1	620	0.063	3.100	108	118
2	200	0.020	2.00	39	49
3	1000	0.020	2.00	71	122
4	200	0.200	2.00	30	31
5	1000	0.200	2.00	220	–
6	200	0.020	5.00	37	47
7	1000	0.020	5.00	51	80
8	200	0.200	5.00	30	31
9	1000	0.200	5.00	168	212

Table S2: Polymerisation in closed vial after Kim et. al.

Time (h)	Thickness (nm)
2	116
4	140
6	165

Table S3: Polymerisation under coverslip with PMDETA, 4 h

#	MeOH fraction	[M] (mM)	[Cu] (mM)	[RA] (mM)	Thickness (nm)
1	0.25	200	0.01	0.50	11
2	0.50	500	0.01	0.50	7
3	0.50	200	0.10	0.50	22
4	0.25	500	0.10	0.50	37
5	0.25	200	0.01	5.00	29
6	0.50	500	0.01	5.00	27
7	0.50	200	0.10	5.00	41
8	0.25	500	0.10	5.00	39
9	0.38	350	0.06	2.75	36

S2 IR spectrum of pSPMA brushes

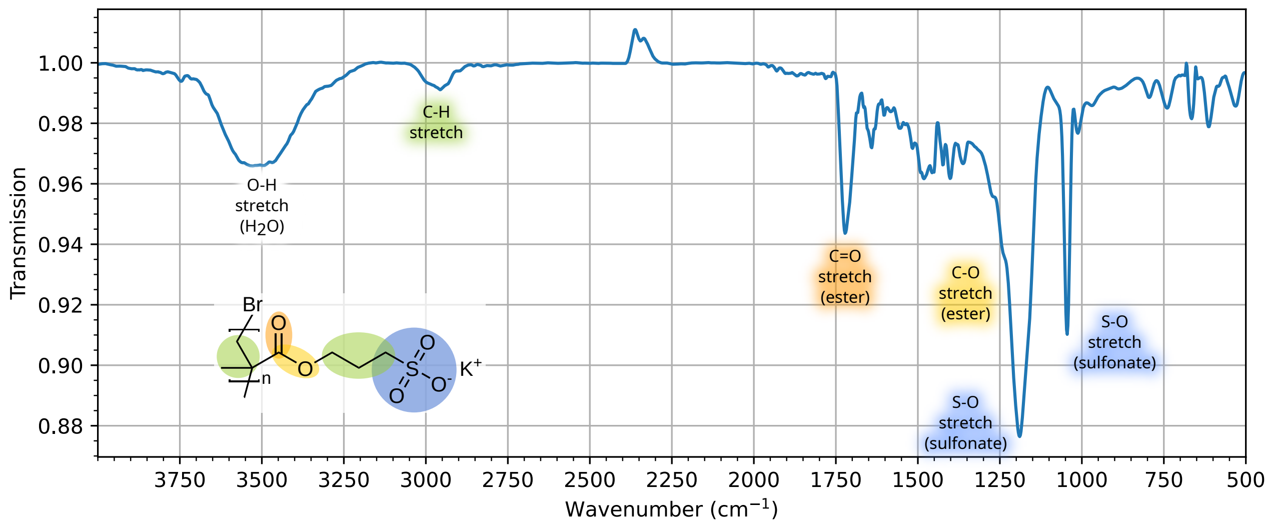

Figure S1: IR spectrum of pSPMA brushes.

S3 AFM images

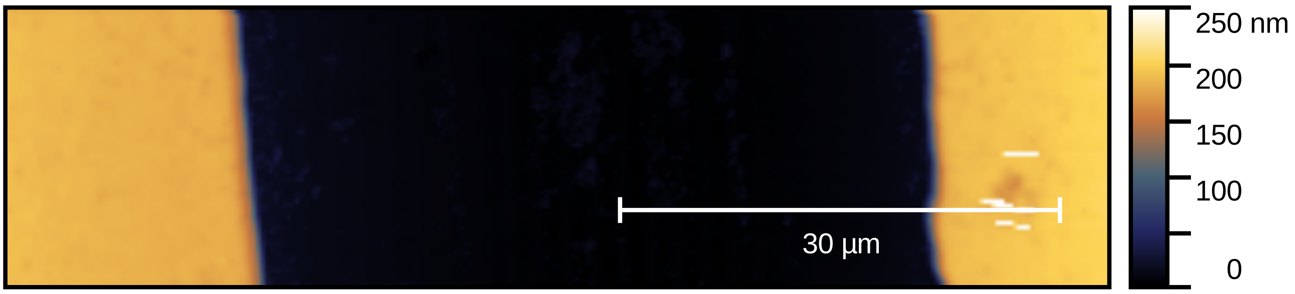

Figure S2: A scratch in the polymer brush, showing the thickness of the brush.

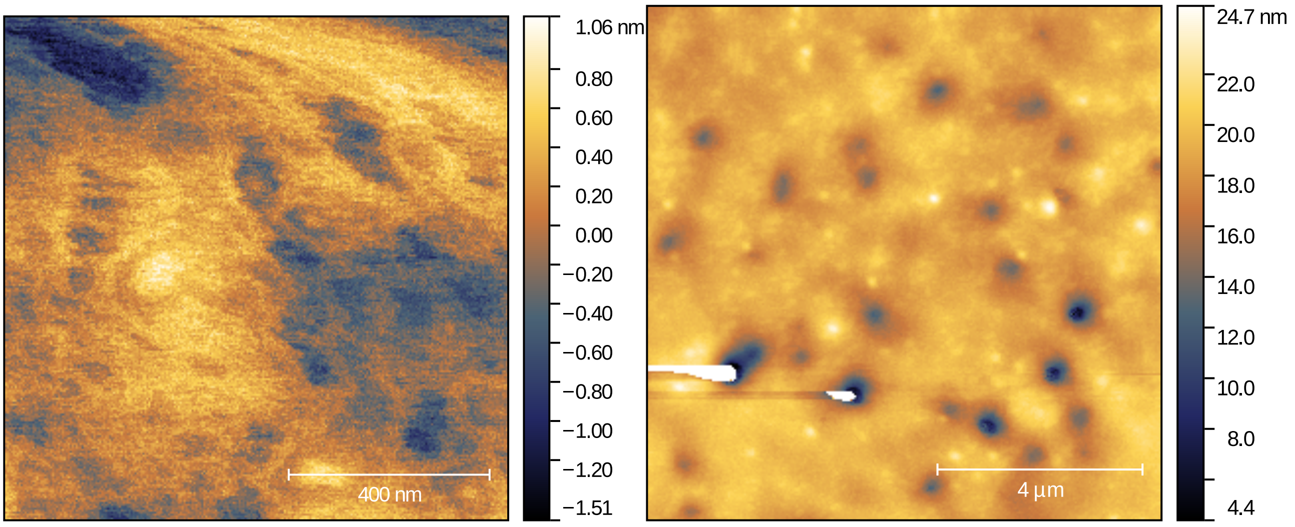

Figure S3: Surface morphology of the polymer brush, 1x1 µm (**left**), and 10x10 µm (**right**).

S4 Optical microscopy images

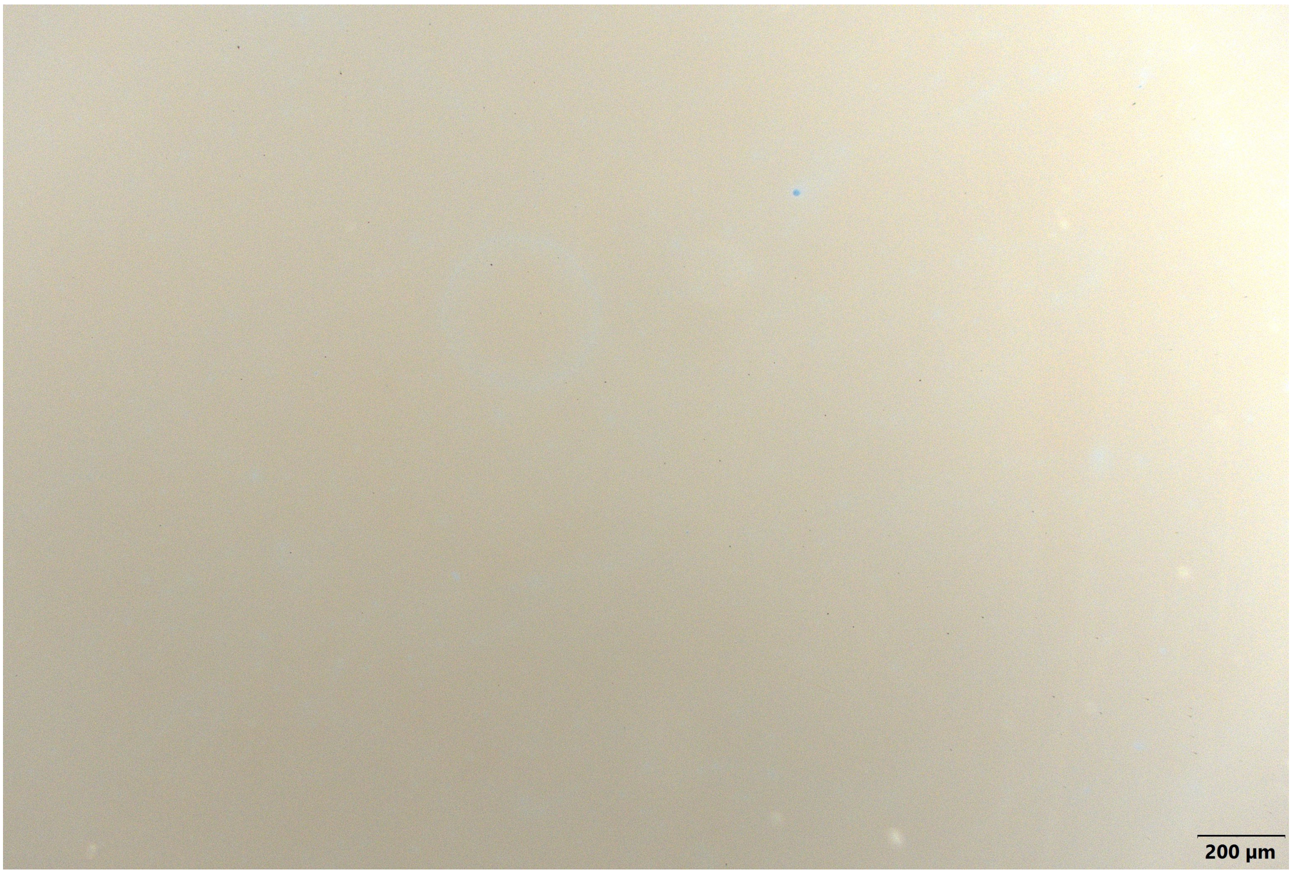

Figure S4: Homogenous center of a brush.

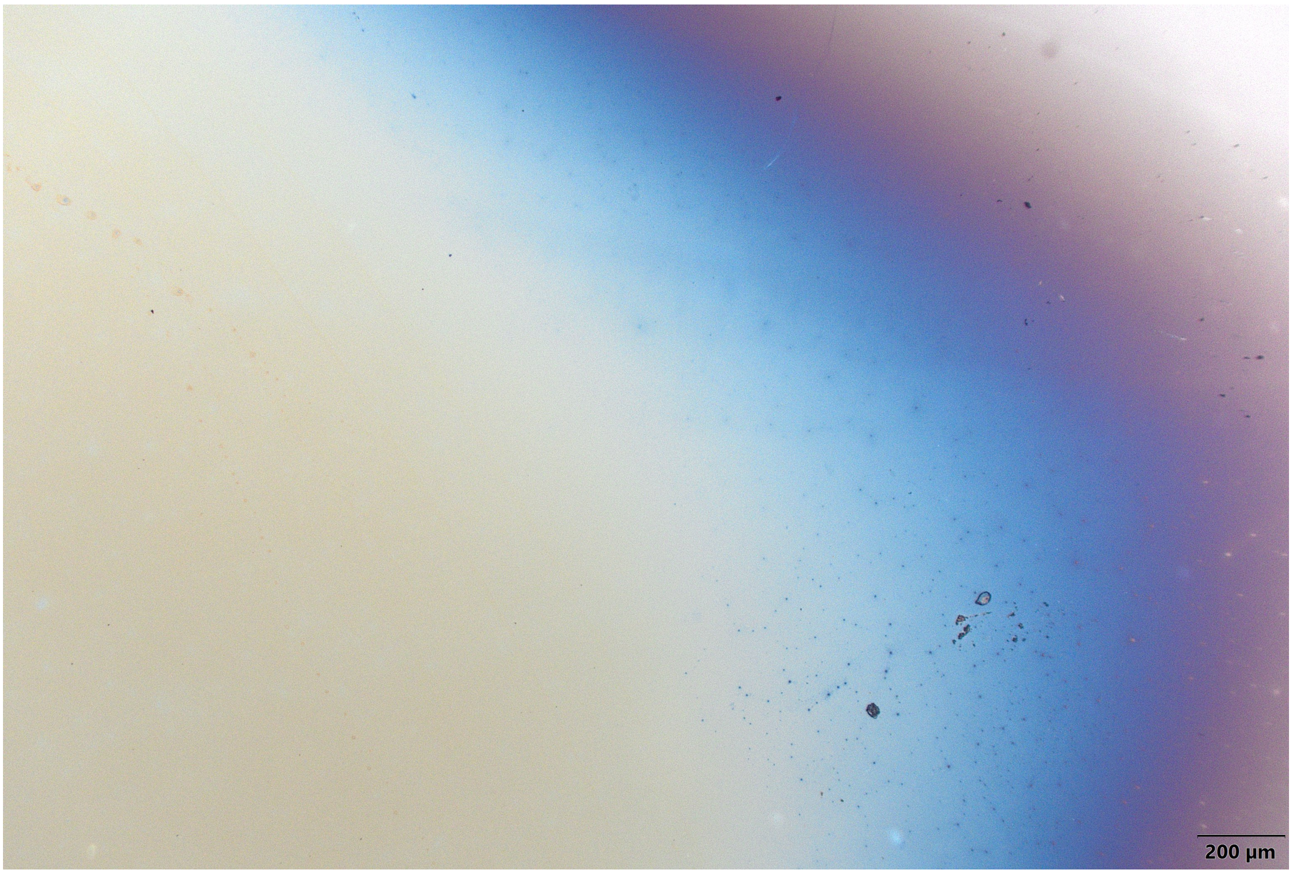

Figure S5: Thickness gradient near the edge of a brush.

S5 Hazy brushes

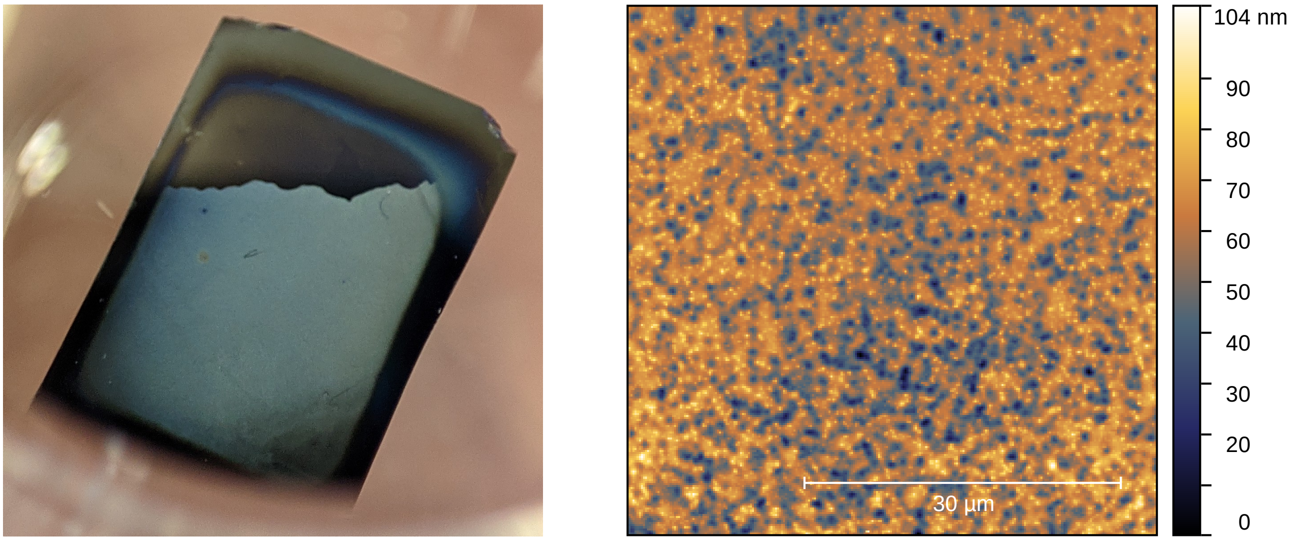

Figure S6: Optical appearance (**left**) and surface morphology (**right**) of a hazy brush.

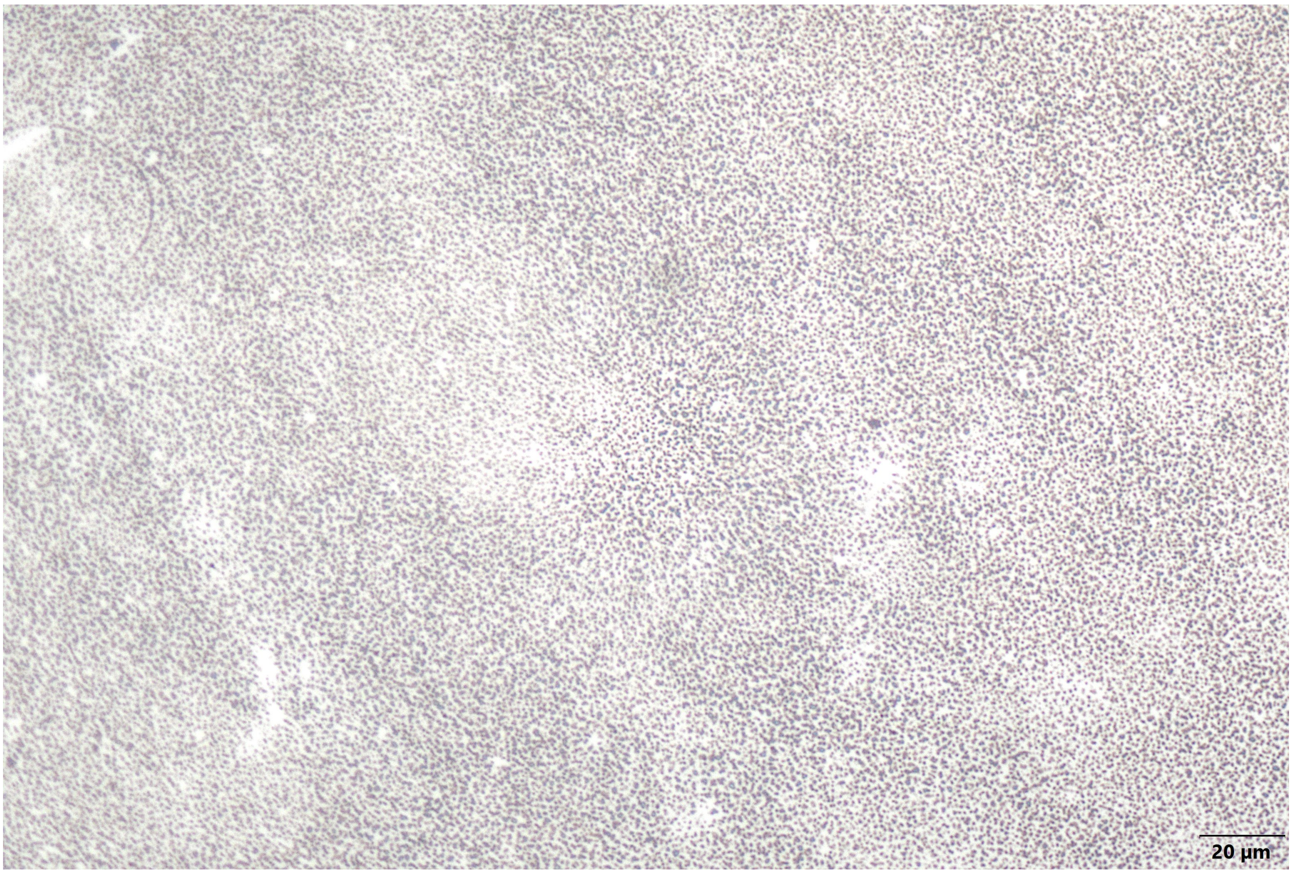

Figure S7: Optical microscopy image of a hazy brush.

S6 0.5 mM AA brushes

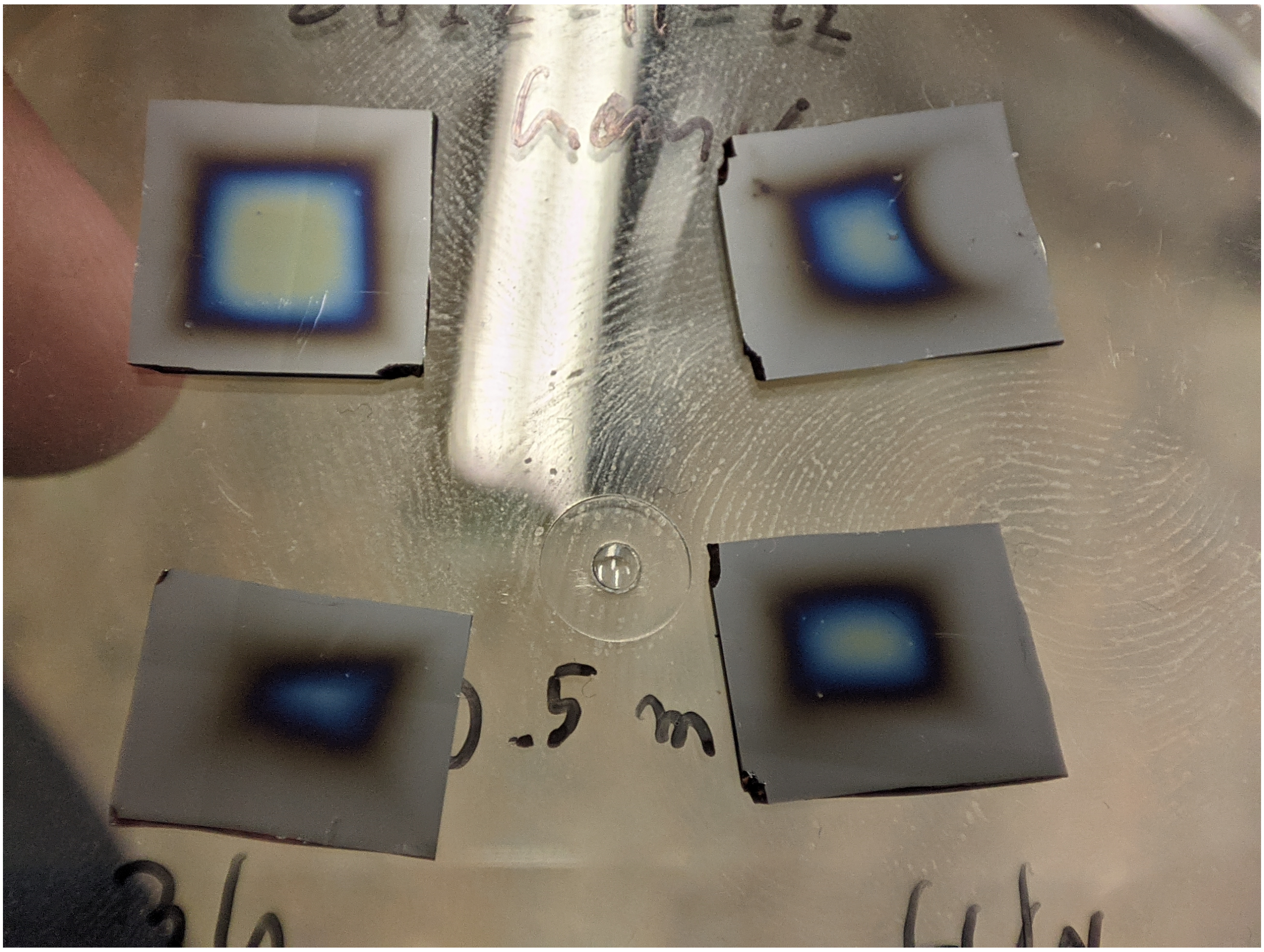

Figure S8: Optical appearance of brushes with 0.5 mM of AA, leading to inconsistent thicknesses and large edge gradients.

S7 Sandwiched wafers

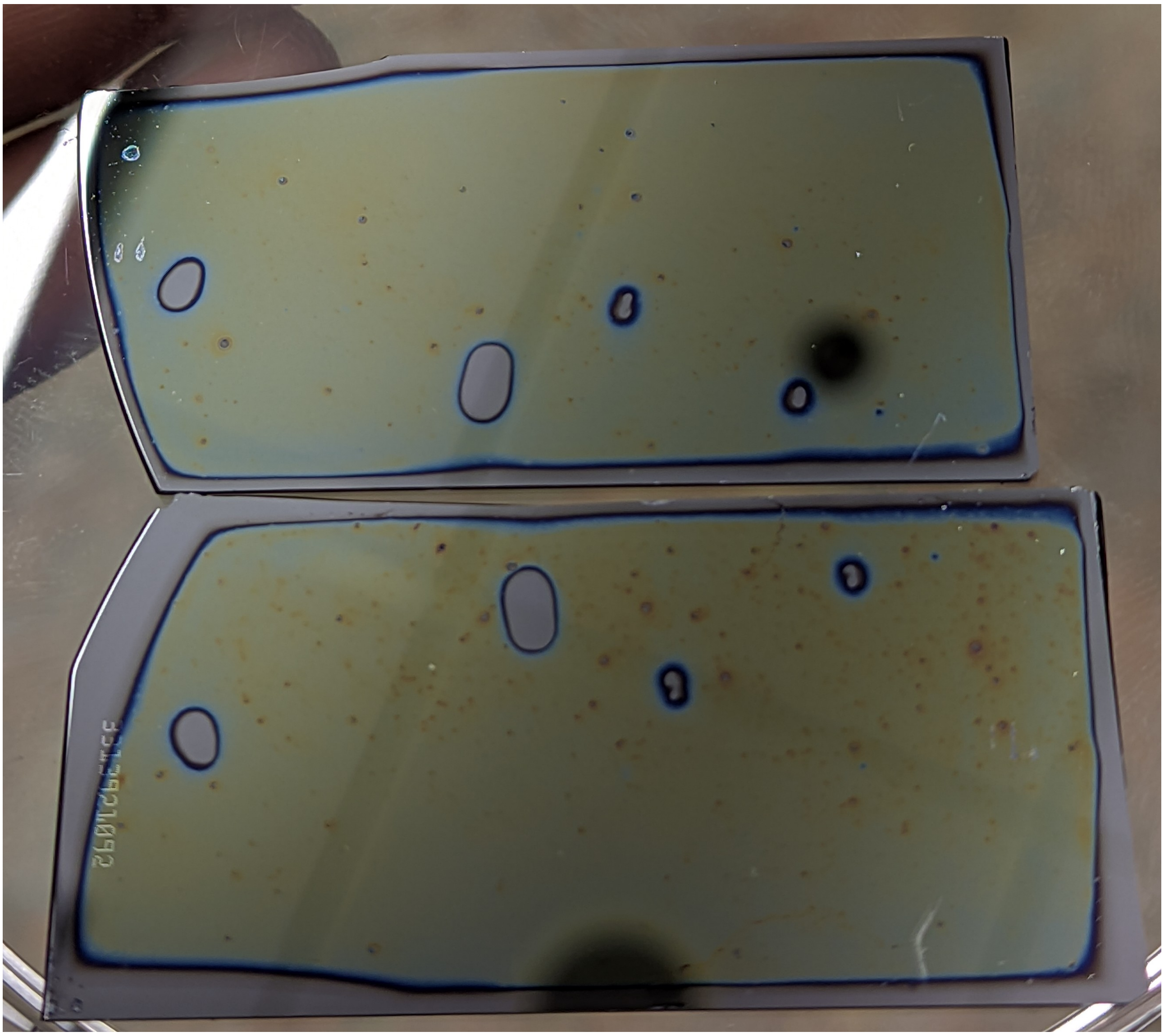

Figure S9: Optical appearance of two brushes obtained by sandwiching two wafers together.

S8 Chain-extended sample

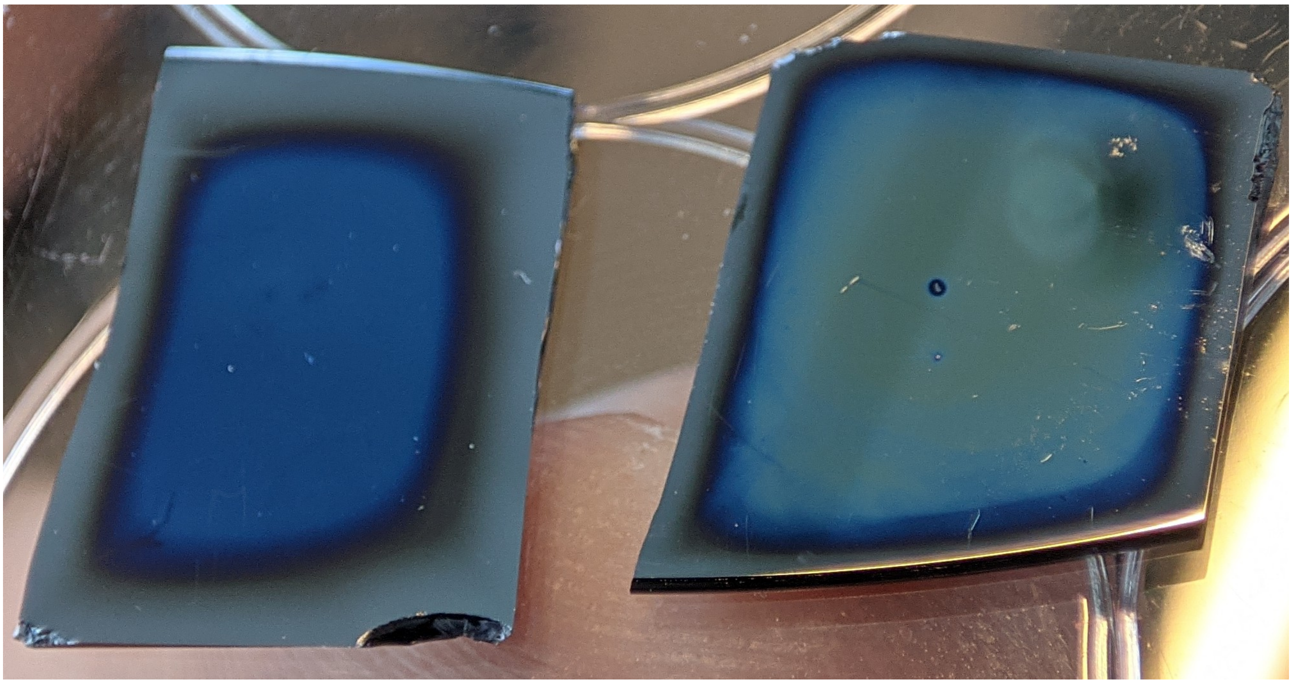

Figure S10: Optical appearance of a brush with a single polymerisation of 1 h (left), and a sample polymerised 3x 1 h (right).
